# Retinoids in cancer chemoprevention and therapy: Meta-analysis of randomized controlled trials

**DOI:** 10.3389/fgene.2022.1065320

**Published:** 2022-11-09

**Authors:** Shuting Chen, Qinchao Hu, Xiaoan Tao, Juan Xia, Tong Wu, Bin Cheng, Juan Wang

**Affiliations:** ^1^ Hospital of Stomatology, Sun Yat-Sen University, Guangzhou, China; ^2^ Guangdong Provincial Key Laboratory of Stomatology, Guangzhou, China; ^3^ Guanghua School of Stomatology, Sun Yat-Sen University, Guangzhou, China

**Keywords:** retinoids, vitamin A, cancer, treatment, prevention

## Abstract

Retinoids, natural and synthetic derivatives of vitamin A, have many regulatory functions in human body, including regulating cellular proliferation, differentiation, apoptosis. Moreover, retinoids have been used successfully for the treatment of certain malignancies, especially acute promyelocytic leukemia (APL) in adults and neuroblastoma in children. However, retinoids have not yet been translated into effective systemic treatments for most solid cancers. Some recent studies have shown that retinoids promote tumorigenesis. Therefore, we performed this meta-analysis to systematically evaluate the efficacy of retinoids in the chemoprevention and treatment of cancers. We performed literature search of several electronic databases, including PubMed, Embase and Cochrane Library from 2000 January to 2021 November. Various outcomes were applied to investigate the potential of retinoids for prevention and treatment of cancers. The primary outcomes in this study were disease recurrence and clinical response. The secondary outcomes included overall survival (OS), cancer development, disease progression and event-free survival. We identified 39 randomized controlled trials with 15,627 patients in this study. Our results showed that lower recurrence rate and better clinical response were obtained in retinoids treated patients with cancer or premalignancy as compared with control. The differences were statistically significant (RR = 0.85, 95% CI = 0.74–0.96, *p* = 0.01; RR = 1.24, 95% CI = 1.03–1.49, *p* = 0.02, respectively). Retinoids treatment was not associated with improvement in overall survival, cancer development, disease progression or event-free survival. Subgroup analysis conducted based on cancer type showed that patients benefited from retinoids treatment in APL, renal cell carcinoma, hepatocellular carcinoma, lung cancer, Kaposi sarcoma, and complete hydatidiform mole. No significant therapeutic effect was noted in head and neck cancer, acute myeloid leukemia (AML), melanoma, breast cancer, bladder cancer, cervical intraepithelial neoplasia (CIN) or cervical carcinoma. Subgroup analysis based on tumor classification demonstrated that retinoids group obtained a lower recurrence rate and better clinical response than control group in solid cancers. In conclusion, clinical application of retinoids was associated with reduction in disease recurrence and improvement in clinical response, illustrating that retinoids play a key role in cancer prevention and therapy. Further research is needed to broaden the utility of retinoids in other types of cancers.

**Systematic Review Registration:** PROSPERO, identifier CRD42022296706.

## Introduction

Retinoids are a family of signaling molecules in humans and they function *via* retinoic acid receptors (RARs) and retinoid X receptors (RXRs). Retinoids cannot be synthesized by mammals, they can only be metabolized and converted from vitamin A. Vitamin A can be absorbed from animal food sources and plants. In animal products, vitamin A exists in the form of retinyl palmitate initially, and then dietary retinyl palmitate is converted to retinol in the intestinal lumen (Harrison, 2005). Retinol ultimately metabolizes to retinoic acid and its derivatives, known as retinoids. Retinoids consist of the natural and synthetic analogues of vitamin A. There are many different types of retinoids, such as all trans retinoic acid (ATRA), 13-cis retinoic acid (13 cRA), etretinate, and fenretinide. They can be classified into three generations regarding the structures and properties. Now the fourth generation is under research with less toxicity and greater efficacy (Khalil et al., 2017).

Retinoids regulate a wide range of biological processes, including cell proliferation and differentiation, apoptosis, immune function, and embryonic development (Tang and Gudas, 2011). Retinoids play vital roles in regulating skin functions, including epidermal keratinization, differentiation and proliferation. Owing to these effects, they have been used to treat skin diseases such as acne vulgaris, psoriasis and photoaging, and show positive efficacy (Cosio et al., 2021).

The synthesis and metabolism of retinoids are aberrant in many human tumors, including oral cavity, skin, bladder, kidney and breast, suggesting that retinoids are closely related to the development and progression of cancer. Lecithin-retinol acyltransferase (LRAT) is an important enzyme of retinoid metabolism, and it increases retinol uptake. Abnormally low intracellular concentrations of retinoids and decreased LRAT expression have been confirmed in various types of cancers including oral cavity, bladder, skin, prostate and breast cancers (Tang and Gudas, 2011). Reduced protein levels of recombinant aldehyde dehydrogenase 1 family, member A2 (ALDH1A2), a key player in the retinoic acid (RA) pathway and retinoid metabolism, have been reported in human prostate and breast cancer. The upregulated expression of CYP26A1, an enzyme responsible for RA oxidation, has been proven to promote the progression of human primary breast cancer (Kim et al., 2005).

Previous studies have proven the potential antitumor effects of retinoids through inducing cell differentiation and inhibiting cell proliferation. Differentiation therapy with ATRA has shown great success in treating acute promyelocytic leukemia (APL) (Ni et al., 2019). Clinical trials using retinoids exerted beneficial therapeutic effects in solid tumors such as breast cancer, cervical cancer, renal cancer, head and neck cancer, basal cell skin cancer, and prostate cancer, but the therapy response to retinoids was often limited to a small part of treated patients (Tang and Gudas, 2011). However, a recent study has shown that retinoids promote tumorigenesis by suppressing the immune system (Devalaraja et al., 2020). Another study demonstrated that the treatment effects of retinoids were not predictable, due to their immune promoting or inhibiting actions, suggesting the complex roles of retinoids in innate and adaptive immunity (Larange and Cheroutre, 2016). The results of published studies are conflicting, and the potential effect of retinoids on prevention and therapy of cancer remains unclear. Thus, this updated meta-analysis will include more eligible RCTs, especially the new studies, to systematically evaluate the effect of retinoids in cancer prevention and therapy.

## Materials and methods

Our meta-analysis was performed according to the PRISMA statement and Cochrane Collaboration Tool. The protocol of our research was registered at the PROSPERO (CRD42022296706).

### Search strategy and selection criteria

We performed literature search of several electronic databases, including PubMed, Embase and Cochrane Library from 2000 January to 2021 November. We also searched ClinicalTrials.gov to further identify relevant registered randomized controlled trials (RCTs). Language and study region were not restricted in the search strategy. The search formula consists of three major parts: retinoids, cancer or premalignancy and RCT filter. Literature search was performed using following MeSH headings in the title or abstract: (retinoic OR retinoid* OR “retinoid derivative*”) AND (neoplasm* OR cancer* OR carcino* OR tumo* OR premalignan* OR precancerous).

We included only these RCTs that reported extractable data on retinoids as treatment or prevention in patients with tumors or precancerous lesions, whereas review, meta-analysis, case report, editorials and conference papers were excluded. All these RCTs compared the efficacy of retinoids and no retinoids (defined as placebo or no treatment).

### Data extraction and quality assessment

Two investigators (SC and QH) performed literature screening independently. Extracted data included author, year of publication, country, subject age, cancer type, intervention method, treatment duration, follow-up duration, sample size, sex ratio, and outcome. Any discrepancies and disagreements were resolved by discussion and consensus with the senior investigators (JW and BC). The methodological quality and internal validity of individual trials were independently assessed by two investigators using the Cochrane Collaboration’s risk of bias tool. There were seven assessment components, each of which could be rated as high risk, low risk or unclear risk according to the criteria.

The primary outcomes in this study were disease recurrence and clinical response. Disease recurrence was defined as relapse of disease after treatment completion. Clinical response was assessed by clinicians in terms of relief or recovery of the disease, including complete resolution and partial resolution. The secondary outcomes were overall survival (OS), cancer development, disease progression and event-free survival. Overall survival was defined as the proportion of patients who were alive from randomization to the end of follow-up period. Cancer development referred to neoplasm events, which included new cancer development, second primary tumor (SPT) and malignant transformation. Disease progression refers to the progression and deterioration of the neoplasia confirmed by histologic examination. Event-free survival was characterized by the proportion of patients who are alive free of neoplasm events.

### Statistical analysis

Review manager 5.4 and Stata 16.0 software were used to perform statistical analysis. We present the results using risk ratios (RR) with 95% confidence intervals (CI) to represent the estimated efficacy of the intervention. I^2^ statistic was applied to evaluate the heterogeneity between studies. Heterogeneity was considered high if I^2^ was greater than 50%. If heterogeneity was high, sensitivity analysis was performed by excluding studies one by one to find out the potential source of heterogeneity. L’Abbe plot was also used to detect the heterogeneity between studies. Random-effect model was applied to synthesize the data. Subgroup analysis was performed based on cancer type and tumor classification. Funnel plot asymmetry and Egger’s test were used to detect potential publication bias. A two-tailed *p* < 0.05 was considered statistically significant.

## Results

### Studies characteristics

**FIGURE 1:**
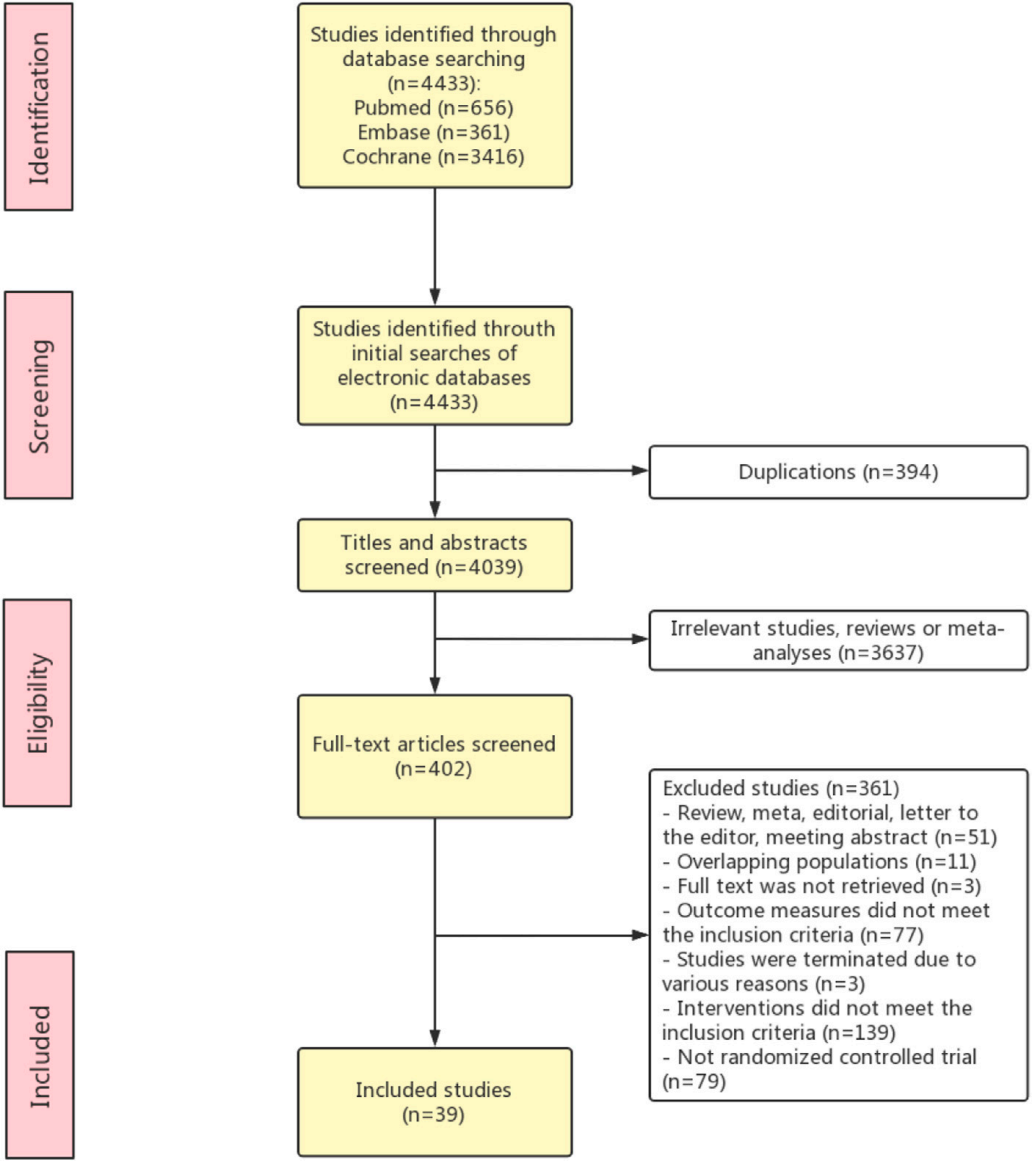
PRISMA flow diagram of study identification.

The literature searching and screening process is presented in Figure 1. Thirty-nine RCT studies with a total of 15,627 patients were included in this meta-analysis (Fenaux et al., 2000; Kohler et al., 2000; Motzer et al., 2000; van Zandwijk et al., 2000; Bodsworth et al., 2001; Follen et al., 2001; Lippman et al., 2001; Bernstein et al., 2002; George et al., 2002; Robinson et al., 2002; Atzpodien et al., 2004; Fosså et al., 2004; Ruffin et al., 2004; Shen et al., 2004; Toma et al., 2004; Aass et al., 2005; Chiesa et al., 2005; Perry et al., 2005; Richtig et al., 2005; Takai et al., 2005; Khuri et al., 2006; Scardina et al., 2006; Veronesi et al., 2006; Burnett et al., 2007; Sabichi et al., 2008; Decensi et al., 2009; Weinstock et al., 2009; Andrijono and Muhilal, 2010; Arrieta et al., 2010; Burnett et al., 2010; Lee et al., 2010; Rao et al., 2011; Kadakia et al., 2012; Weinstock et al., 2012; Douer et al., 2013; Nazha et al., 2013; Shinagawa et al., 2014; Aviles et al., 2015; Okita et al., 2015b; Bhatia et al., 2017; Sanusi, 2019). The characteristics of the included studies are shown in Table 1. The quality assessment of the included studies was shown in Supplementary Figure S1. Sixteen studies were conducted in North America, 14 studies were conducted in Europe, six studies were proceeded in Asia, and 3 studies were proceeded in Oceania. Among the 39 included studies, 14 studies described the overall survival, and others evaluated the cancer development (*n* = 14), clinical response (*n* = 13), disease recurrence (*n* = 9), disease progression (*n* = 4), and event-free survival (*n* = 3).

**TABLE 1:** Characteristics of included studies.

Author year	Country	Age	Cancer type	Intervention	Treatment duration	Follow-up duration	Sample size	Female,%	Outcome
Zandwijk 2000	Italy	Median age 61 years	Patient with head and neck cancer or lung cancer treated	Retinyl palmitate vs. no retinyl palmitate	2 years	49 months	1290/1283	13	OS, SPT
Khuri (2006)	United States	Mean ± SD 60.8 ± 10.9	Patients who had been treated for stage I or II HNSCC	Isotretinoin (30 mg/d) vs. placebo	3 years	4 years	590/600	21	OS, SPT
Perry (2005)	Australia	Unclear	Patients with their first head and neck squamous cell carcinoma treated	Isotretinoin (1.0 mg/kg/d) vs. isotretinoin (0.5 mg/kg/d) vs. placebo	3 years	Unclear	54/48/49	24	SPT
Bhatia (2017)	United States	Median age 60 years	Treated stage I/II SCC of the oral cavity, oropharynx, hypopharynx, larynx	13-Cis Retinoic Acid vs. placebo	2 years	5 years	91/85	22	OS, SPT
Lippman (2001)	United States	Mean ± SD 64.2 ± 8.8	Treated stage I non-small-cell lung cancer	Isotretinoin (30 mg/day) vs. placebo	3 years	3.5 years	589/577	43	OS, SPT, recurrence
Veronesi 1999/2006	Italy	Mean ± SD 51 ± 7.6/51 ± 7.8	Surgically removed stage I breast cancer or ductal carcinoma *in situ*	Fenretinide orally (200 mg/day) vs. no treatment	5 years	14.6 years	1432/1435	100	SPT, recurrence
Toma (2004)	Italy	Unclear	Squamous cell carcinoma of head and neck	Isotretinoin vs. control	unclear	39 months	126/126	Unclear	OS, disease progression
Sabichi (2008)	United States	Median age 70.9/64.0	Resected non-muscle-invasive bladder cancer	Fenretinide (200 mg/day orally) vs. placebo	12 months	15 months	70/67	0	Recurrence
Kohler (2000)	United Kingdom	Children	Advanced neuroblastoma after chemotherapy	13-Cis retinoic acid 0.75 mg/kg/day vs. placebo	4 years	59 months	88/87	Unclear	EFS
Kadakia (2012)	United States	Mean ± SD 68.2 ± 9.48	Treated nonmelanoma skin cancers (NMSCs)	Acitretin 25 mg orally 5 days per week vs. placebo	2 years	Unclear	35/35	37	New NMSC development
Takai (2005)	Japan	Unclear	Treated Hepatocellular Carcinoma (HCC)	Acyclic Retinoid 600 mg/d vs. placebo	48 weeks	7 years	44/45	Unclear	OS, SPT
Weinstock (2012)	United States	Mean age 71 years	Patients with history of BCC or SCC of the skin	Topical 0.1% tretinoin cream vs. control	3 years	3.5 years	566/565	3	OS, cancer development
Douer (2013)	United States	Median age 36	Untreated acute promyelocytic leukemia (APL)	ATRA 45 mg/m^2^/d orally vs. observation	1 year	11.8 years	101/117	34	Relapse
George (2002)	Australia	Mean ± SD 56.7 ± 9.5 (Group1)/52.2 ± 10.2 (Group2)	Patients with history of non-melanoma skin cancers	Acitretin 25 mg orally, once daily vs. drug-free	2 years	Unclear	23/23	22	Cancer development
Dencensi (2009)	Italy	Mean ± SD 46.2 ± 5.2	Premenopausal Women at risk of breast cancer	fenretinide 200 mg/d vs. placebo	2 years	5.5 years	59/58	100	Breast cancer events
Shinagawa (2014)	Japan	Median age 48	Patients with newly diagnosed APL	ATRA or tamibarotene	2 years	1 year	135/134	47	Relapse, EFS
Okita 2014	Japan	Unclear	Treated hepatitis C-related hepatocellular carcinoma	peretinoin 600 mg/day, peretinoin 300 mg/day, or placebo	96 weeks	3 years	124/126/127	36	OS, EFS, recurrence
Richtig (2005)	Austria	Mean ± SD 52.6 ± 13.8	Resected melanoma in stage IIA and IIB	IFN + isotretinoin vs. IFN + placebo	2 years	5 years	206/201	47	OS, disease progression
Rao (2011)	United States	Median age 72	Postmenopausal women with hormone receptor-positive breast cancer resected	Tamoxifen + fenretinide vs. tamoxifen + placebo	5 years	5 years	206/213	100	Recurrence, SPT
Aass (2005)	Norway	Median age 60	Progressive metastatic renal cell carcinoma treated	IFN-α-2a + 13-cRA vs. IFN-α-2a alone	13–15 weeks	3 months	159/161	31	Relapse, OS
Motzer (2000)	United States	Median age 60	Advanced renal cell carcinoma	13-cis-retinoic acid + IFN-α-2a vs. IFN-α-2a	60 months	Unclear	139/145	33	OS, CR
Atzpodien (2004)	Germany	Median age 59	Irresectable metastatic renal cell carcinoma	13 cRA + IFN-α-2a/IL-2/IV-FU vs. IFN-α-2a/IL-2/IV-FU	8 weeks	Unclear	146/132	25	OS, CR
Aviles (2015)	Mexico	Median age 63	Patients with refractory/relapsed, cutaneous T-cell lymphoma, with advanced stages	IFN + ATRA vs. IFN + MTX	6 months	12 months	176/201	32	OS
Bernstein (2002)	United States	Mean ± SD 37.5 ± 6.7/41.2 ± 6.7	Patients with AIDS-associated Kaposi sarcoma	ATRA once weekly vs. 3 times weekly	12 weeks	Unclear	30/46	3	CR
Bodsworth (2001)	Australia	Median age 38	Cutaneous AIDS-Related Kaposi Sarcoma	Alitretinoin gel 0.1% twice daily vs. vehicle gel	12 weeks	Unclear	62/72	0	CR
Sanusi (2019)	Indonesia	Mean ± SD 54.0 ± 9.2/47.7 ± 8.2	Locally advanced cervical carcinoma	NAC (cisplatin and paclitaxel) + vitamin A VS. NAC	64 weeks	Unclear	15/15	100	CR
Fosså (2004)	Norway	Median age 60	Progressive, metastatic renal cell carcinoma	IFN vs. IFN + 13-cis retinoic acid	3 months	1 year	53/50	43	CR
Arrieta (2010)	Mexico	Median age 59	Advanced non–small-cell lung cancer	Paclitaxel/cisplatin (P/C) + ATRA vs. P/C + placebo	80–100 days	80–100 days	52/55	45	CR
Burnett 2009	United Kingdom	Median age 45	Acute Myeloid Leukemia	Treatment with ATRA or not	8 years	Unclear	540/535	49	OS, relapse, CR
Burnett (2007)	United Kingdom	Median age 65	Older patients Not Considered Fit for Intensive Treatment with acute myeloid leukemia	Cytarabine (Ara-C) or hydroxyurea with or without ATRA	3 years	Unclear	107/99	44	OS, CR
Nazha 2013	United States	Median age 66	Older patients with acute myeloid leukemia	Intensive chemotherapy with or without ATRA	Unclear	12.5 years	36/34	Unclear	CR
Fenaux (2000)	France	Unclear	Newly diagnosed APL	ATRA + chemotherapy vs. chemotherapy	Unclear	73 months	54/47	Unclear	Relapse, CR
Shen (2004)	China	Median age 32	Newly diagnosed APL	ATRA + As2O3 vs. As2O3	Unclear	18 months	21/20	49	CR
Scardina (2006)	Italy	Unclear	Oral leukoplakia	Isotretinoin 0.18% vs. 0.05% (topical, twice a day)	3 months	10 years	20/20	Unclear	Malignant change
Chiesa (2005)	Italy	Median age 50	Oral leukoplakias	200 mg fenretinide daily vs. no intervention	1 year	5 years	84/86	29	Cancer development
Andrijono (2010)	Thailand	Median age 25	Complete hydatidiform mole	Vitamin A vs. placebo	Unclear	20 mon	32/35	100	Malignant disease
Robinson (2002)	United States	Unclear	HIV-positive women with low-grade squamous intraepithelial lesions of the cervix	Isotretinoin vs. observation	6 months	192 weeks	56/58	100	Disease progression
Ruffin (2004)	United States	Unclear	Women with biopsy-proven CIN II/III	ATRA (0.16%, 0.28%, 0.36%) vs. placebo	4 days	12 weeks	136/38	100	Regression rate
Follen (2001)	United States	Unclear	Biopsy-proven CIN-2/3 (High-Grade SIL)	4-HPR at 200 mg/day vs. placebo	6 months	12 months	20/16	100	CR, disease progression

OS, overall survival; SPT, second primary tumor; EFS, event-free survival; CR, clinical response; ATRA, all trans retinoic acid; IFN, interferon; 13-cRA, 13-cis retinoic acid; IL-2, interleukin-2; IV, intravenous; FU, fluorouracil; MTX, methotrexate; NAC, neoadjuvant chemotherapy; 4-HPR, N-(4-hydroxyphenyl) retinamide.

### Primary outcomes

**FIGURE 2:**
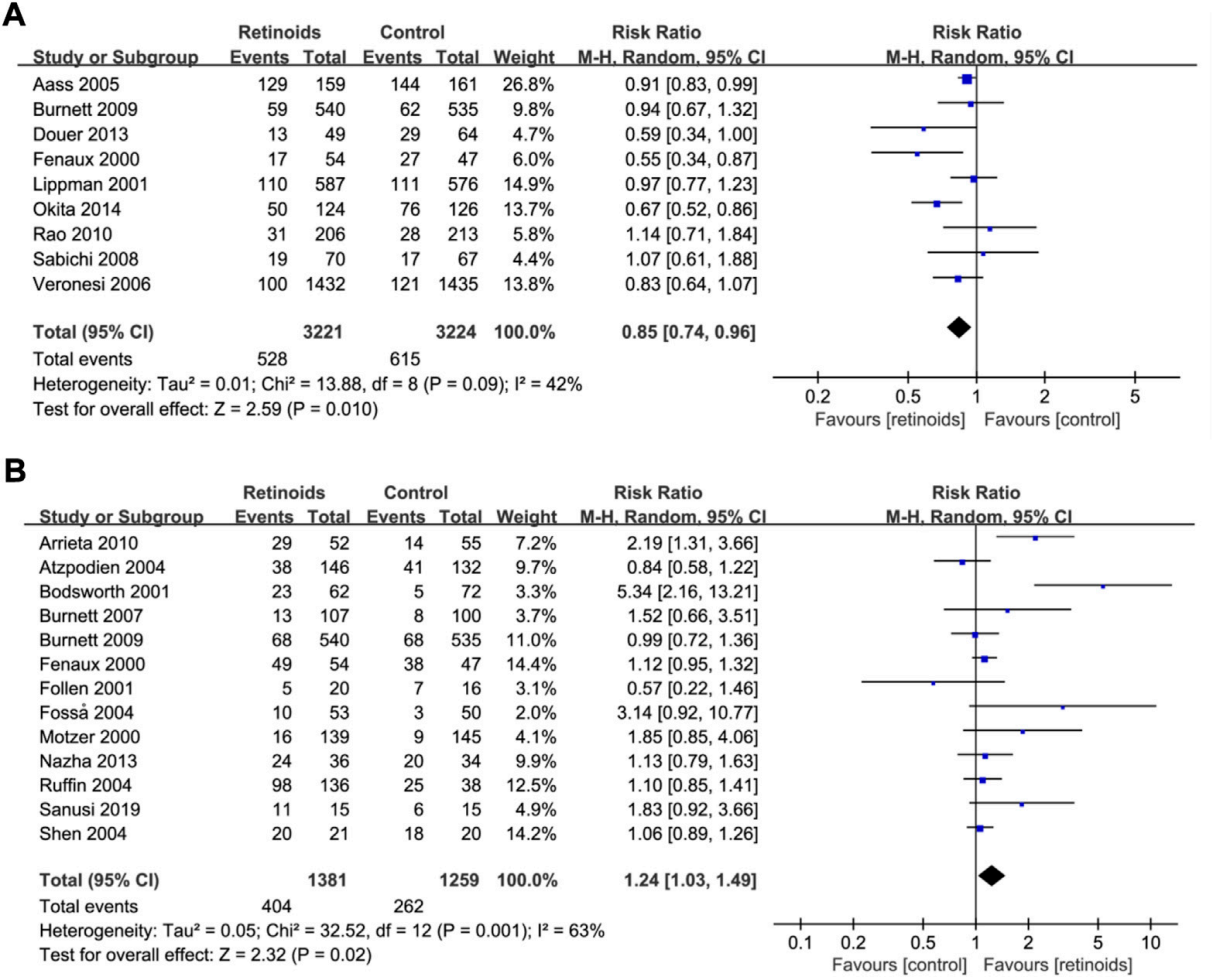
Forest plots showed the effects of retinoids application in cancers or precancerous lesions concerning primary outcomes. **(A)** disease recurrence, **(B)** clinical response. CI, confidence interval.

The association of retinoids and disease recurrence was supplied by nine studies (Fenaux et al., 2000; Lippman et al., 2001; Aass et al., 2005; Burnett et al., 2007; Sabichi et al., 2008; Rao et al., 2011; Douer et al., 2013; Okita et al., 2015a) with 6,445 patients. Data analysis revealed that retinoids usage was correlated with lower recurrence rate compared with control group (RR = 0.85, 95% CI = 0.74–0.96, *p* = 0.01) (Figure 2A). The heterogeneity was acceptable according to the statistic (I^2^ = 42%) (Figure 2A).

Pooled data in 13 studies (Fenaux et al., 2000; Motzer et al., 2000; Bodsworth et al., 2001; Follen et al., 2001; Atzpodien et al., 2004; Fosså et al., 2004; Ruffin et al., 2004; Shen et al., 2004; Burnett et al., 2007; Arrieta et al., 2010; Burnett et al., 2010; Nazha et al., 2013; Sanusi, 2019) with 2,640 patients showed that retinoids group exhibited a better clinical response rate than control group (RR = 1.24, 95% CI = 1.03–1.49, *p* = 0.02) (Figure 2B). Heterogeneity among the above 13 studies was significant (I^2^ = 63%) (Figure 2B).

### Secondary outcomes

**FIGURE 3:**
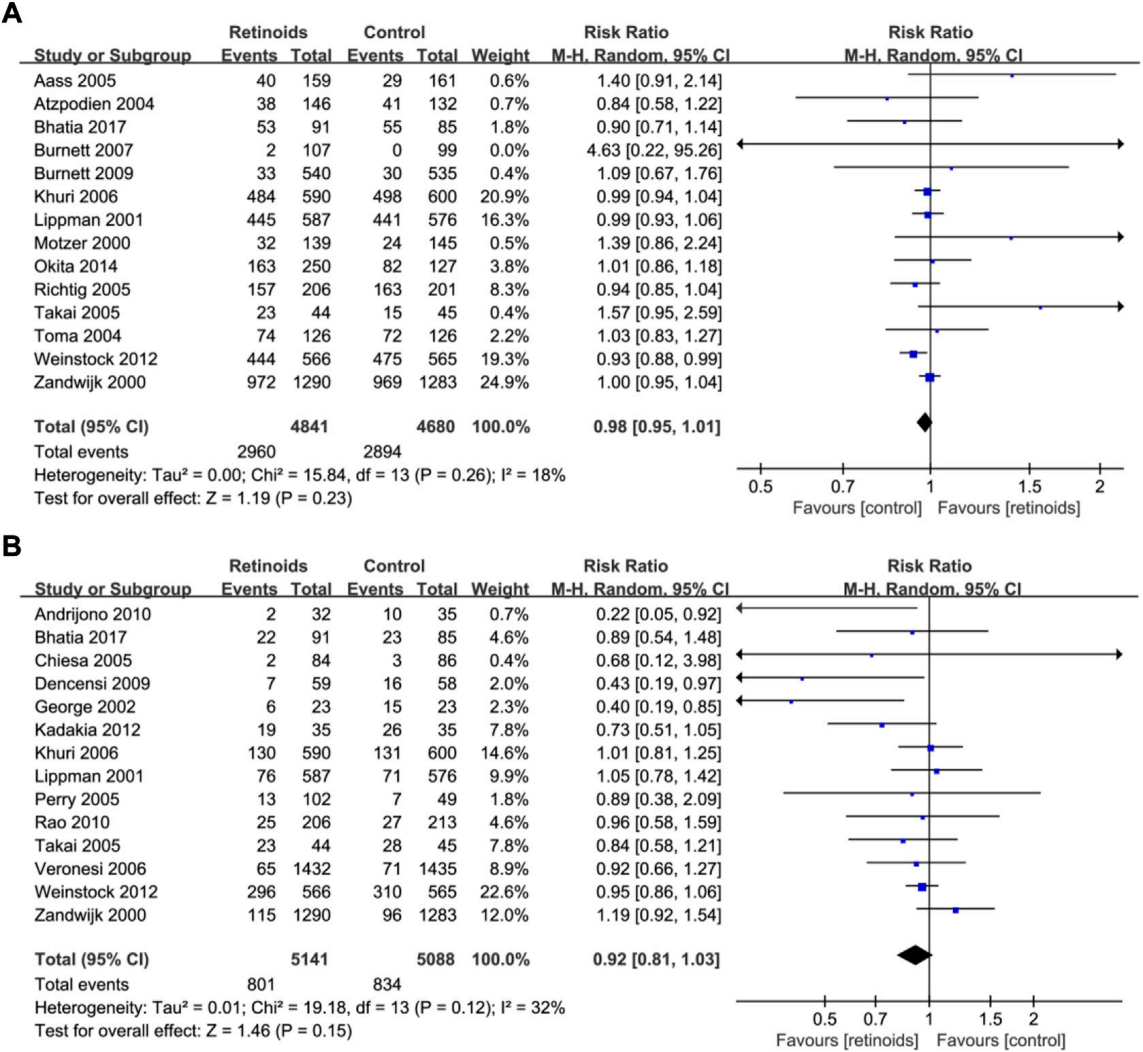
Forest plots showed the effects of retinoids application in cancers or precancerous lesions concerning secondary outcomes. **(A)** overall survival (OS), **(B)** cancer development. CI, confidence interval.

Fourteen studies (Motzer et al., 2000; van Zandwijk et al., 2000; Lippman et al., 2001; Atzpodien et al., 2004; Toma et al., 2004; Aass et al., 2005; Richtig et al., 2005; Takai et al., 2005; Khuri et al., 2006; Burnett et al., 2007; Weinstock et al., 2009; Burnett et al., 2010; Bhatia et al., 2017) with 9,521 patients reported the data of retinoids and overall survival. Synthesized data showed that there was no significant relation between retinoids usage and overall survival (RR = 0.98, 95% CI = 0.95–1.01, *p* = 0.23) (Figure 3A) without obvious heterogeneity (I^2^ = 18%) (Figure 3A).

Fourteen studies (van Zandwijk et al., 2000; Lippman et al., 2001; George et al., 2002; Chiesa et al., 2005; Perry et al., 2005; Takai et al., 2005; Khuri et al., 2006; Veronesi et al., 2006; Decensi et al., 2009; Weinstock et al., 2009; Andrijono and Muhilal, 2010; Rao et al., 2011; Kadakia et al., 2012; Bhatia et al., 2017) with 10,229 patients showed the relationship between retinoids and cancer development. Data analysis showed that there was no significant association between retinoids application and cancer development (RR = 0.92, 95% CI = 0.81–1.03, *p* = 0.15) (Figure 3B). Acceptable heterogeneity was presented (I^2^ = 32%) (Figure 3B).

For the disease progression, four studies (Follen et al., 2001; Robinson et al., 2002; Toma et al., 2004; Richtig et al., 2005) with 798 patients reported that there was no significant relation between retinoids application and disease progression (RR = 1.17, 95% CI = 0.86–1.60, *p* = 0.32) (Supplementary Figure S2) without obvious heterogeneity (I^2^ = 0%) (Supplementary Figure S2).

As for event-free survival, three studies (Kohler et al., 2000; Shinagawa et al., 2014; Okita et al., 2015b) with 556 patients evaluated the relationship between retinoids and event-free survival. No difference was observed statistically between retinoids group and control group based on the pooled data (RR = 0.94, 95% CI = 0.87–1.02, *p* = 0.13) (Supplementary Figure S3). No heterogeneity was found (I^2^ = 0%) (Supplementary Figure S3).

### Sensitivity analysis

Heterogeneity analysis as well as sensitivity analysis of our meta-analysis were conducted mainly based on overall survival as it has the maximum included studies. According to the statistics, heterogeneity was not significant (I^2^ = 18%) (Figure 3A). Furthermore, L’Abbe plot also showed that there was no significant heterogeneity detected (Supplementary Figure S4). However, there is a considerable heterogeneity in the primary outcome clinical response (I^2^ = 63%) (Figure 2B). Sensitivity analysis was performed by excluding studies one by one, and then we found that one RCT (Bodsworth et al., 2001) is the potential origin of heterogeneity. After removing it, the heterogeneity declined to 42%. Whether excluding this RCT or not, similar trends of retinoids treatment in clinical response were observed and it confirmed the robustness of our results (Supplementary Figure S5).

### Subgroup analysis based on cancer type

**FIGURE 4:**
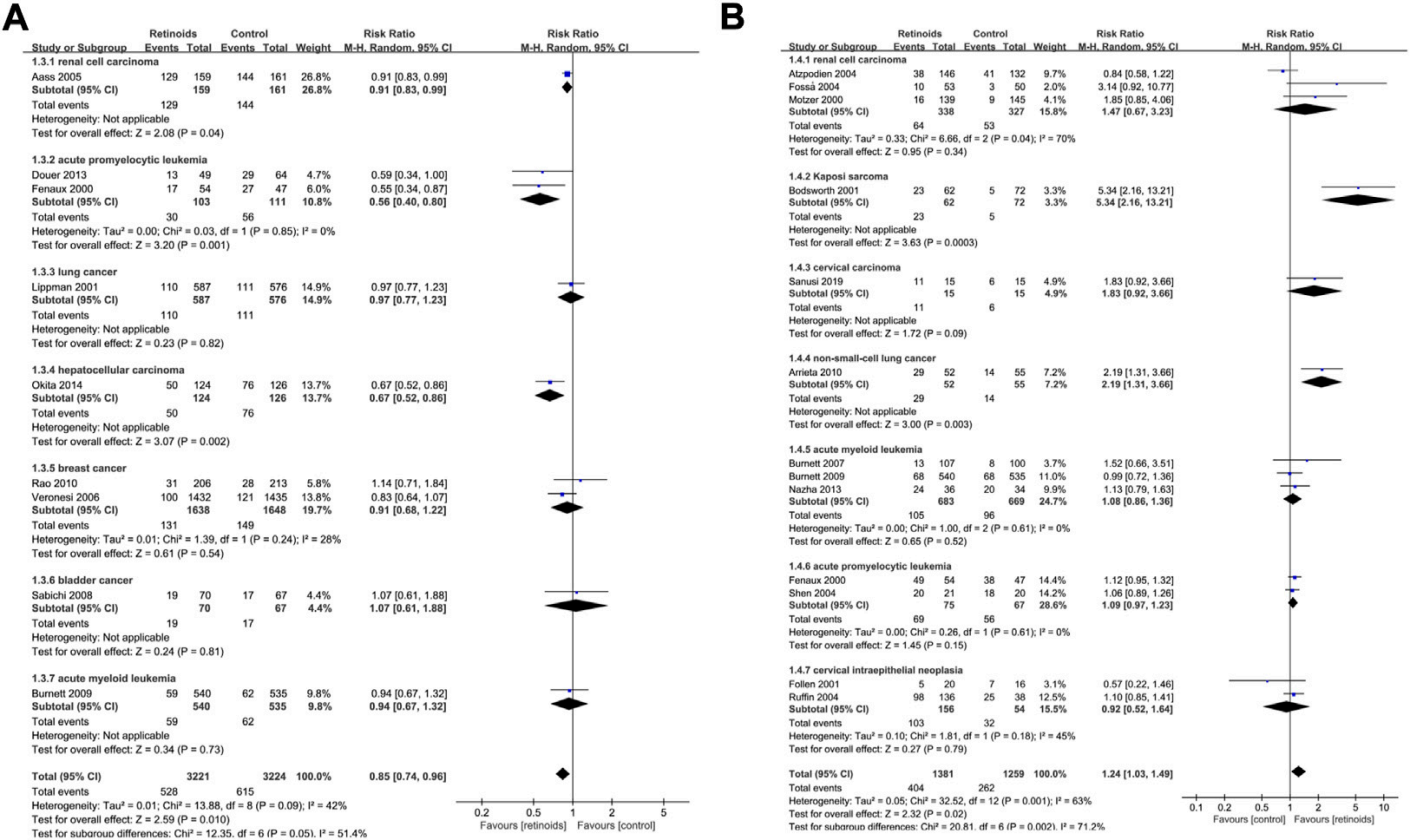
Subgroup analysis based on cancer type described the effects of retinoids application in cancers or precancerous lesions concerning primary outcomes. **(A)** disease recurrence, **(B)** clinical response. CI, confidence interval.

Subgroup analysis based on cancer type of primary outcome disease recurrence showed that retinoids induced lower rate of recurrence in APL (*p* = 0.001), renal cell carcinoma (*p* = 0.04) and hepatocellular carcinoma (*p* = 0.002) (Figure 4A). On the contrary, no significant effect of retinoids was found in lung cancer (*p* = 0.82), breast cancer (*p* = 0.54), bladder cancer (*p* = 0.81) and AML (*p* = 0.73). As for primary outcome clinical response (Figure 4B), higher clinical response rate was detected in Kaposi sarcoma (*p* = 0.0003) and non-small-cell lung cancer (*p* = 0.003) owing to retinoids application. However, no difference was found between retinoids group and control group in renal cell carcinoma (*p* = 0.34), cervical carcinoma (*p* = 0.09), AML (*p* = 0.52), APL (*p* = 0.15), and cervical intraepithelial neoplasia (*p* = 0.48).

**FIGURE 5:**
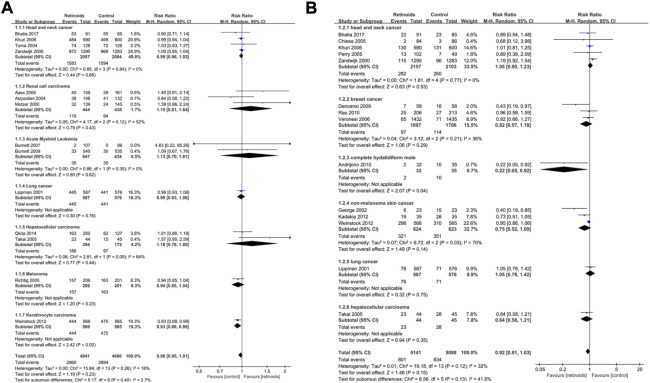
Subgroup analysis based on cancer type described the effects of retinoids application in cancers or precancerous lesions concerning secondary outcomes. **(A)** overall survival (OS), **(B)** cancer development. CI, confidence interval.

As for secondary outcome overall survival (Figure 5A), there was no significant association between retinoids usage and overall survival in head and neck cancer (*p* = 0.66), renal cell carcinoma (*p* = 0.43), AML (*p* = 0.62), lung cancer (*p* = 0.76), hepatocellular carcinoma (*p* = 0.44), and melanoma (*p* = 0.23). However, poorer overall survival was related to retinoids usage in keratinocyte carcinoma (*p* = 0.02). Pooled analysis on secondary outcome cancer development revealed that there was no difference between retinoids group and control group in head and neck cancer (*p* = 0.53), breast cancer (*p* = 0.29), non-melanoma skin cancer (*p* = 0.14), lung cancer (*p* = 0.75), and hepatocellular carcinoma (*p* = 0.35) (Figure 5B). Nevertheless, retinoids group showed a lower rate of cancer development in complete hydatidiform mole (*p* = 0.04).

### Subgroup analysis based on tumor classification

The included cancers in this study can be divided into two general classifications: solid cancer and hematology malignancy. For primary outcome disease recurrence, subgroup analysis based on tumor classification demonstrated that retinoids group obtained a lower rate of recurrence than control group in solid cancers (RR = 0.88, 95% CI = 0.78–0.99, *p* = 0.04) (Table 2). The difference was statistically significant (*p* = 0.04). However, as for hematology malignancy, no significant difference was found between retinoids group and control group concerning disease recurrence (RR = 0.70, 95% CI = 0.48–1.02, *p* = 0.06) (Table 2). Similar findings were also seen in primary outcome clinical response. Pooled data revealed that retinoids usage was correlated with a better clinical response rate in solid cancers (RR = 1.56, 95% CI = 1.03–2.36, *p* = 0.04) (Table 2) and exhibited statistically significant difference (*p* = 0.04). In hematology malignancy, there was no significant correlation between retinoids application and clinical response (RR = 1.09, 95% CI = 0.98–1.21, *p* = 0.11) (Table 2).

**TABLE 2:** Subgroup analysis of tumor classification.

Outcome	Subgroup	Study number	Sample size	RR (95%CI)	*p*-value	Heterogeneity	
						I^2^ (%)	*p*-value
Disease recurrence	Overall	9	6445	0.85 (0.74–0.96)	0.01	42	0.09
	Tumor classification						
	Solid cancer	6	5156	0.88 (0.78–0.99)	0.04	31	0.2
	Hematology malignancy	3	1289	0.70 (0.48–1.02)	0.06	55	0.11
Clinical response	Overall	13	2640	1.24 (1.03–1.49)	0.02	63	0.001
	Tumor classification						
	Solid cancer	8	1146	1.56 (1.03–2.36)	0.04	75	0.0002
	Hematology malignancy	5	1494	1.09 (0.98–1.21)	0.11	0	0.87
Overall survival	Overall	14	9521	0.98 (0.95–1.01)	0.23	18	0.26
	Tumor classification						
	Solid cancer	12	9139	0.98 (0.95–1.02)	0.3	23	0.22
	Hematology malignancy	2	382	1.02 (0.42–2.47)	0.96	15	0.28

As for secondary outcome overall survival (OS), synthesized data showed that there was no difference statistically between retinoids group and control group in either solid cancers (RR = 0.98, 95% CI = 0.95–1.02, *p* = 0.30) (Table 2) or hematology malignancy (RR = 1.02, 95% CI = 0.42–2.47, *p* = 0.96) (Table 2).

### Publication bias

We analyzed publication bias in studies regarding overall survival using funnel plot asymmetry. No obvious asymmetry was observed, indicating that there was no apparent publication bias, which was confirmed by Egger’s test (*p* = 0.0784) (Supplementary Figure S6).

## Discussion

Retinoids are a family of signaling molecules in human beings and they play vital roles in cell differentiation, proliferation, and apoptosis. The synthesis and metabolism of retinoids are impaired in various cancers (Tang and Gudas, 2011). It is critically necessary to clarify the correlation between retinoids and carcinogenesis risk. Many previous preclinical and clinical investigations revealed the great potential of retinoids in the chemoprevention and treatment of several types of cancers. However, a few recent studies have reported that retinoids promote tumorigenesis (Larange and Cheroutre, 2016; Devalaraja et al., 2020). Therefore, we performed this meta-analysis of RCTs to systematically evaluate the effects of retinoids on cancers. We investigated the association between disease recurrence, clinical response, overall survival, cancer development, disease progression, event-free survival, and retinoids application in patients with tumors or precancerous lesions. A more detailed subgroup analysis was also conducted based on cancer type. Our results showed that lower recurrence rate and better clinical response were obtained in retinoids group as compared with placebo group or no treatment group. However, retinoids application was not correlated with overall survival, cancer development, disease progression or event-free survival in patients with cancer or premalignancy.

According to our research, retinoids showed preventive and therapeutic effectiveness in certain cancers. Retinoids application lowered the rate of malignant transformation in patients with complete hydatidiform mole. Moreover, retinoids reduced tumor recurrence in renal cell carcinoma, APL and hepatocellular carcinoma. Better clinical response was obtained in Kaposi sarcoma and non-small-cell lung cancer in retinoids group. One previous study confirmed the benefit of retinoids in prolonging disease-free survival in patients with hepatocellular carcinoma (Chu et al., 2010). As for lung cancer, Yu et al. (2015) reported that dietary vitamin A intake could reduce the risk of lung cancer, however two other studies demonstrated the ineffectiveness of vitamin A in the treatment or prevention of lung cancer (Fritz et al., 2011; Cortés-Jofré et al., 2020). The studies mentioned above included cohort studies or case-control studies while our study was strictly restricted to RCTs. Thus, our meta-analysis could provide more reliable evidence to clarify the effectiveness of retinoids in cancer chemoprevention and therapy.

Subgroup analysis based on cancer type indicated that no significant difference was observed in any outcome between retinoids and control group in patients with head and neck cancer, AML, melanoma, breast cancer, bladder cancer, cervical intraepithelial neoplasia (CIN) or cervical carcinoma. Our study was consistent with the previous research reported by He et al. (2018), which showed that vitamin A derivatives intake had no effects on the survival of breast cancer patients. However, Fulan et al. (2011) claimed that both the total intake of retinol and vitamin A could reduce breast cancer risk. These contradictory studies suggested that retinol might have preventive effect rather than therapeutic effect on breast cancer. Similar contradictory findings were also seen in cervical diseases and bladder cancer. One study showed that retinoids had no effect in preventing the progression of CIN (Helm et al., 2013), while another study demonstrated that vitamin A intake was inversely associated with cervical cancer risk (Zhang et al., 2012). High vitamin A intake was also associated with low bladder cancer risk (Tang et al., 2014). As shown above, the antitumor efficacy of retinoids is limited to several types of cancers, which may be attributed to retinoid resistance. Patients treated with retinoids often exhibit or develop resistance to this therapy. The molecular mechanisms of this resistance remain incompletely understood and the following factors have been confirmed involved: increased expression of RA binding proteins, new mutations in retinoid receptors and the pharmacologic alteration in RA metabolism (Chlapek et al., 2018). Strategies to overcome retinoid resistance include combination therapy as well as the alternative use of non-classical retinoids. Combination of retinoids and interferon-α showed partial or complete response in lymphoid malignancy and preclinical studies indicated that non-classical retinoids can function as tumor suppressors in human cancers (Tang and Gudas, 2011). Well-designed comprehensive studies focused on combination retinoid therapy are recommended.

Subgroup analysis based on tumor classification revealed that retinoids group obtained a lower recurrence rate and better clinical response than control group in solid cancers. But in hematology malignancy, there was no significant difference between retinoids group and control group. We further analyze the possible causes. In our meta-analysis, the included hematology malignancies were consisted of APL and AML. Previous studies have shown that retinoids treatment acquires great success in APL but not in AML (Ni et al., 2019). These findings were also consistent with our subgroup analysis based on cancer type. Thus, the ineffectiveness of retinoids in hematology malignancy may be attributed to the synthesized analysis integrating APL and AML. All in all, our work indicated the potential chemoprevention effect of retinoids for the treatment of solid cancers. Well-designed and large-scaled studies are warranted to confirm our findings.

It should be noted that one RCT included in this study showed that patients with keratinocyte carcinoma obtained a worse overall survival in tretinoin-treated group as compared with control group. However, further analysis by the investigator revealed that the increased mortality in tretinoin-treated group could hardly be attributed to retinoids application, owing to lack of additional supportive evidence and failing to specify the causes of deaths such as smoking status (Weinstock et al., 2009). A recent preclinical study also reported the tumorigenic role of retinoids in fibrosarcoma and melanoma *via* inhibiting immune system (Devalaraja et al., 2020). However, the finding of this study is still lack of support data from clinical research. More large-scale comprehensive RCTs are warranted to figure out whether retinoids have tumorigenic role.

The main limitation of our study may be heterogeneity in interventions and outcomes among studies, such as different categories of retinoids, drug dosage, application pattern, and frequency. The heterogeneity was not significant in any outcome except for clinical response. By excluding RCTs one by one, we found that one RCT study (Bodsworth et al., 2001) is the potential source of heterogeneity. This RCT demonstrated that the alitretinoin group showed a significant higher clinical response rate than control group, which made it distinct from other included studies. This remarkably high response rate of this RCT study may be the main cause of heterogeneity in clinical response. We searched more studies to clarify this problem and found that an approximate clinical response rate of alitretinoin was shown in a recent systematic review comprising three clinical trials and three case reports (Htet et al., 2022). Besides, alitretinoin is Food and Drug Administration-approved medication for Kaposi sarcoma. All these studies demonstrated that alitretinoin had a significant efficacy for the treatment of Kaposi sarcoma. Moreover, synthesized analysis showed that whether excluding or retaining the study (Bodsworth et al., 2001), the result of retinoids treatment in clinical response remained unchanged and this confirmed the robustness of our results.

## Conclusion

Our meta-analysis of RCTs revealed the positive preventive and therapeutic effects of retinoids in several malignancies. Retinoids usage reduced disease recurrence and improved clinical response. Combination therapy containing retinoids may be promising cancer therapy in the future.

## Data Availability

The original contributions presented in the study are included in the article/Supplementary Material, further inquiries can be directed to the corresponding authors.
