# Supplementary material for: Retinoids in cancer chemoprevention and therapy: Meta-analysis of randomized controlled trials
Source: Front Genet. 2022 Nov 9;13:1065320. doi: 10.3389/fgene.2022.1065320 (PMC9681997; doi:10.3389/fgene.2022.1065320)
Supplement: Supplementary file 1 [file DataSheet1.docx]

Supplementary Figures


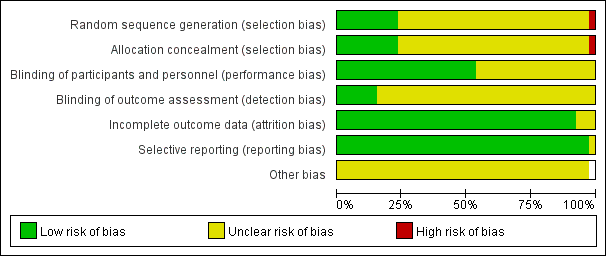


Figure S1. Quality assessment of included studies using Cochrane Collaboration Tool.


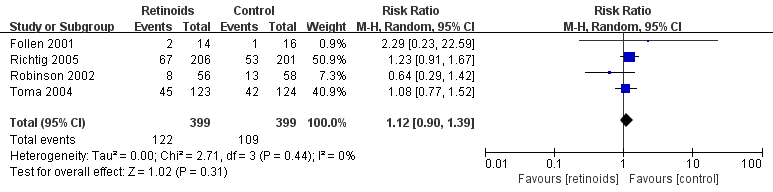


Figure S2. Forest plot showed the relationship between retinoids application and disease progression.


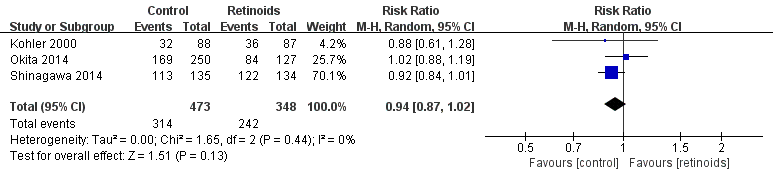


Figure S3. Forest plot showed the relationship between retinoids application and event-free survival.


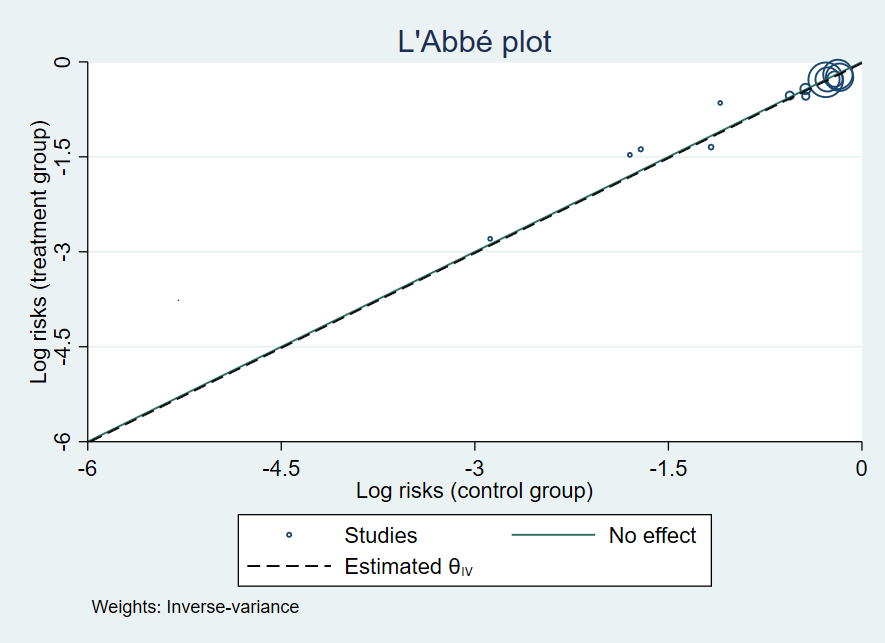


Figure S4. L’Abbe plot detected the potential source of heterogeneity.


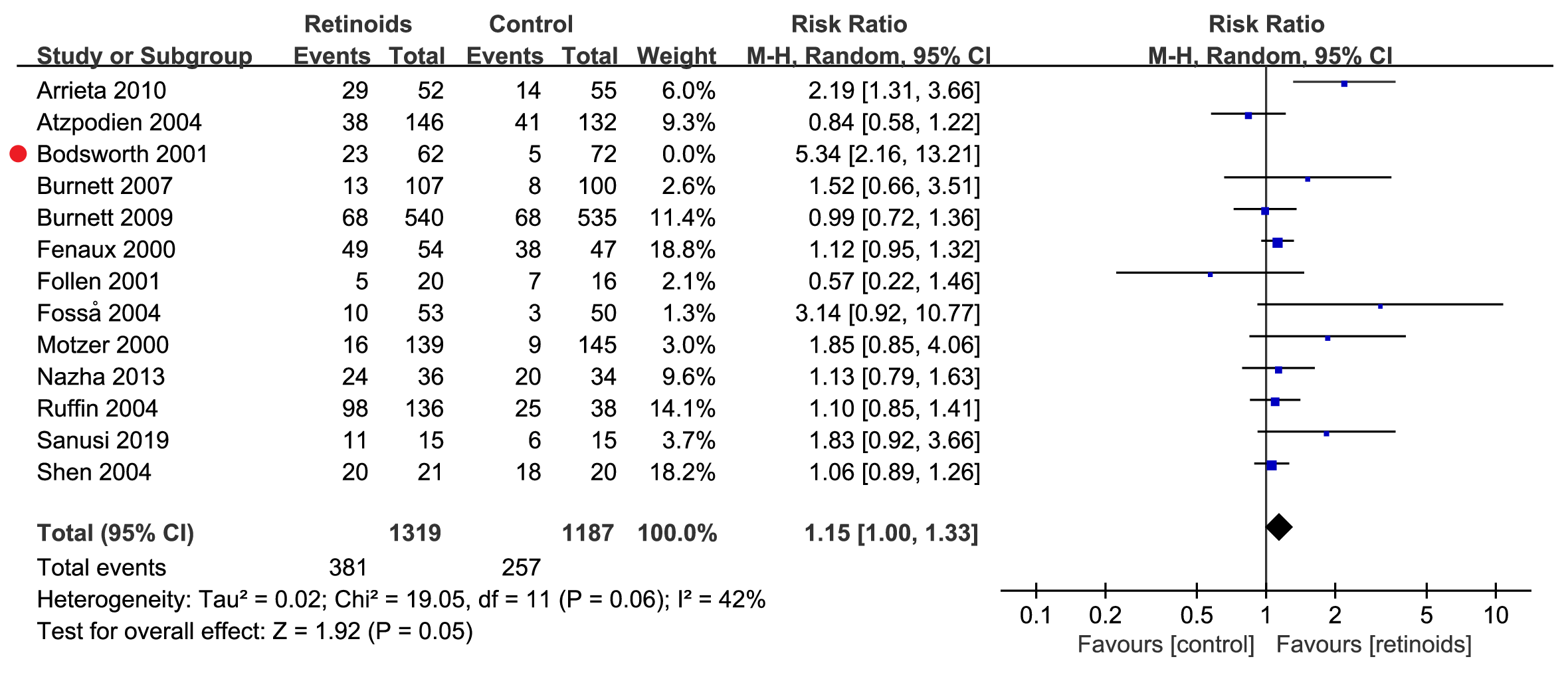


**(B)**


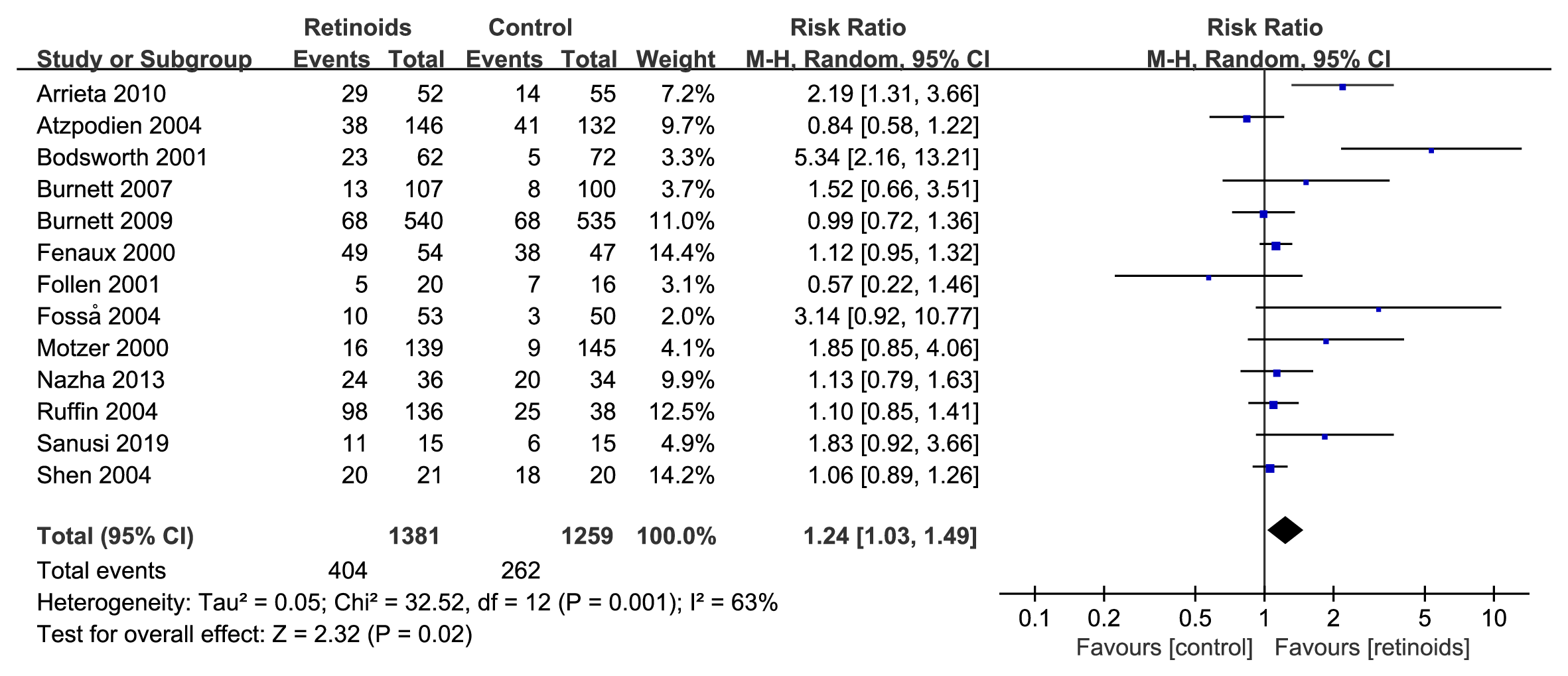


**(A)**

Figure S5. Sensitivity analysis of primary outcome clinical response was performed by excluding studies one by one. (A) The heterogeneity was significant (I^2^ = 63%). (B) The potential source of heterogeneity (Bodsworth 2001) was detected and excluded. The heterogeneity was acceptable (I^2^ = 42%). Similar trends of retinoids treatment in clinical response were observed and it confirmed the robustness of our results.


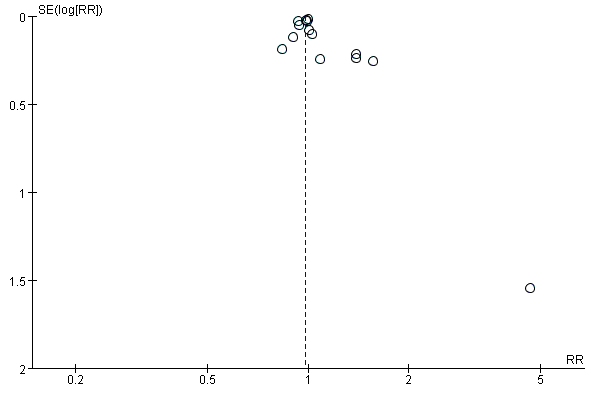


Figure S6. Publication bias assessed studies regarding overall survival using funnel plot asymmetry.
